# A stimulus-responsive, in situ-forming, nanoparticle-laden hydrogel for ocular drug delivery

**DOI:** 10.1007/s13346-018-0504-x

**Published:** 2018-03-05

**Authors:** Maryam Kabiri, Syed H. Kamal, Sandip V. Pawar, Protiva R. Roy, Maziar Derakhshandeh, Ujendra Kumar, Savvas G. Hatzikiriakos, Sazzad Hossain, Vikramaditya G. Yadav

**Affiliations:** 1 Department of Chemical and Biological Engineering, The University of British Columbia, Vancouver, BC Canada; 2 Evonik Transferra Nanosciences, Burnaby, BC Canada; 3 Department of Chemical and Petroleum Engineering, University of Calgary, Calgary, AB Canada; 4 Faculty of Pharmaceutical Sciences, The University of British Columbia, Vancouver, BC Canada; 5 InMed Pharmaceuticals, Inc., Vancouver, BC Canada; 6 School of Biomedical Engineering, The University of British Columbia, Vancouver, BC Canada

**Keywords:** Cannabinoids, Glaucoma, Hydrogel, Nanoparticles, Synthetic biology, Switchable rheology

## Abstract

Most medications targeting optic neuropathies are administered as eye drops. However, their corneal penetration efficiencies are typically < 5%. There is a clear, unmet need for novel transcorneal drug delivery vehicles. To this end, we have developed a stimulus-responsive, in situ-forming, nanoparticle-laden hydrogel for controlled release of poorly bioavailable drugs into the aqueous humor of the eye. The hydrogel is formulated as a composite of hyaluronic acid (HA) and methylcellulose (MC). The amphiphilic nanoparticles are composed of poly(ethylene oxide) (PEO) and poly(lactic acid) (PLA). Experimental design aided the identification of hydrogel composition and nanoparticle content in the formulation, and the formulation reliably switched between thixotropy and temperature-dependent rheopexy when it was tested in a rheometer under conditions that simulate the ocular surface, including blinking. These properties should ensure that the formulation coats the cornea through blinking of the eyelid and facilitate application of the medication as an eye drop immediately prior to the patient’s bedtime. We subsequently tested the efficacy of our formulation in whole-eye experiments by loading the nanoparticles with cannabigerolic acid (CBGA). Our formulation exhibits over a 300% increase in transcorneal penetration over control formulations. This work paves the way for the introduction of novel products targeting ocular diseases to the market.

## Introduction

The space enclosed by the cornea and iris is referred to as the anterior chamber of the eye. The base of the cornea extends into a tissue called the ciliary body, and this interface is aligned by a spongy tissue called the trabecular meshwork [1]. Epithelial cells in the ciliary body secrete a thin, gelatinous fluid known as the aqueous humor that permeates through the trabecular meshwork to fill the anterior chamber. As freshly secreted aqueous humor makes its way into the chamber, older fluid drains out, also through the meshwork. The constant circulation of aqueous humor through the trabecular meshwork is central to eye hygiene and health. Likewise, the posterior chamber of the eye, which is enclosed by the lens and the retina, is filled with a viscous, jelly-like fluid called the vitreous humor. Inadequate or obstructed drainage of the aqueous humor through the trabecular meshwork, which is the pathophysiology of glaucoma, increases the fluid pressure within the anterior chamber, which subsequently propagates to the posterior chamber of the eye [2]. The increased intraocular pressure exacts a toll on the basal membrane of the retina, thinning the mesh-like tissue in this region and damaging the head of the optic nerve housed therein by inducing apoptotic degeneration of the nerve’s ganglion cells.

Chronic optic neuropathies such as glaucoma are the leading causes of blindness worldwide [3]. It is estimated that nearly 80 million people will be affected by these conditions by 2020 [4]. Most neuroprotective medications for treating optic neuropathies are administered as eye drops. However, since less than 5% of the drug in eye drops actually penetrates the cornea [5, 6], the dosage of drug in the formulation is typically much higher than what is required, which implies that eye drops are fairly expensive to manufacture. Additionally, once the neuroprotective drug penetrates the cornea and lens, it must then diffuse through the aqueous and vitreous humors before it can interact with receptors on the surface of retinal ganglion cells. These barriers to mass transport further lower the efficacy of drugs administered as eye drops. There is considerable room for improvement for drug formulations for ophthalmological applications, in particular, new and effective delivery vehicles for neuroprotective drugs.

Our therapeutic strategy leverages the proven potential of cannabinoids to confer neuroprotection to ganglion cells [7]. Although the role of cannabinoids in treating glaucoma is well understood, no such products currently exist in the market. Current glaucoma remedies work by lowering intraocular pressure either by inhibiting carbonic anhydrase in the eye, or reducing the production of aqueous humor by the ciliary epithelial cells, or by increasing fluid drainage through the trabecular meshwork [8]. Likewise, devices that provide a conduit for the intraocular fluid to drain into the nasolacrimal system have also been tested for the treatment of glaucoma [9]. The nasolacrimal drainage system discharges tear fluid from the external eye into the nasal cavity and plays key roles in maintenance of eye hygiene and clearance of drugs. Neuroprotection as a therapeutic strategy has not hitherto been pursued owing to great difficulties associated with the targeted delivery of cannabinoids to the base of retina and the poor bioavailability of these molecules [10]. Previous attempts for topical delivery of cannabinoids to human ocular tissues were limited to using mineral oil [11] or cyclodextrins [12–15]. However, these formulations are either cytotoxic or irritate the ocular tissue [16]. The development of a superior drug delivery vehicle would surely pave the way to market for non-invasive, cannabinoid-based neuropathic pain relievers for treating glaucoma and other chronic optic neuropathies.

To this end, we have developed a stimulus-responsive, in situ-forming, nanoparticle-laden hydrogel for spatiotemporal and dosage-controlled release of poorly bioavailable cannabinoids into the aqueous humor of the eye. The hydrogel is formulated as a composite of hyaluronic acid (HA) and methylcellulose (MC). The former is an anionic polysaccharide, and the latter is a water-soluble, hydrophobic derivative of cellulose. Both materials are biocompatible and highly mucoadhesive and have been recognized as safe by the FDA [17, 18]. In order to enhance the flux of the drug through the cornea and sustain its release for a prolonged duration, we also employed nanoparticles as the drug carriers. The nanoparticles (NPs) are composed of poly(ethylene oxide) (PEO) and poly(lactic acid) (PLA) and are loaded with cannabigerolic acid (CBGA), a molecule that is a close mimic of the cannabinoids that have been approved for use in glaucoma medication. Additionally, we synthesized the CBGA by expressing the cannabinoid biosynthetic pathway in *Escherichia coli*, and the drug-loaded NPs were synthesized using nanoprecipitation. Since we desired rapid gelation of the formulation upon contact with the ocular surface and formation of a uniform, unintrusive coating over the cornea that can be positioned by blinking of the eyelid, we implemented a factorial design of experiments in order to identify the constitution of the formulation that allows it to switch between temperature-dependent rheopexy (thickening) and thixotropy (thinning). Parameters that were investigated include the concentration of HA and MC, the size of the NPs, and the drug loading, among others. The optimized formulation has a sol-gel transition temperature of roughly 32 °C, which is comparable with that of the ocular surface, and successfully transitioned between shear thinning and temperature thickening when it was tested in a rheometer under conditions that simulated the ocular surface. Finally, we tested the efficacy of our formulation in whole-eye experiments using porcine eyeballs. Our work seamlessly combines product design, synthetic biology, polymer rheology and analysis of mass transport within ocular tissue, and the formulation that we have developed exhibits over a 300% increase in transcorneal penetration over control formulations. Moreover, the nanoparticle-laden hydrogel can be packaged as a liquid, which permits easy dosing and manufacturability. The current study paves the way to the market for cannabinoid-based drugs, as well as combination therapies with existing intraocular pressure-lowering pharmaceuticals.

## Experimental section

### Chemicals

Sodium hyaluronate (MW 752 kDa) was purchased from Lifecore Biomedical LLC (Chicago, IL). Methylcellulose (MC A15 PREM LV) was obtained as a gift from Dow Chemical (Midland, MI). PEO-b-PLA (5.0-b-23.0) was purchased from Polymer Source Inc. (Montreal, QC). Carboxy-functionalized poly(styrene) NPs bearing a range of sizes were purchased from Phosphorex (Hopkinton, MA). Olivetolic acid (OA) was synthesized as described previously [19], and all reagents were purchased from Sigma Aldrich Canada (Oakville, ON). Geranyl pyrophosphate (GPP) was also purchased from Sigma Aldrich Canada. Water was distilled and deionized using Millipore Milli-RO 10 Plus and Milli-Q UF Plus (Bedford, MA) to a final resistivity of 18 MΩ. Simulated tear fluid (STF) was prepared in accordance with previously published protocols [20]. All organic solvents used in the current study were of HPLC grade and were purchased from Sigma Aldrich Canada.

## Methods

### Preparation of NP-laden hydrogels

MC was initially dissolved in half of the volume of deionized (DI) water that is required for the sample under investigation. For instance, if the final concentration of MC that was required in the sample was 10 g/L, 10 g of MC was first dissolved in 500 mL of DI water. The DI water was close to boiling (~ 90 °C) when it was added to MC, and the mixture was agitated until the solids were completely immersed. The lower critical solution temperature (LCST) of MC in water ranges between 40 and 50 °C, which implies that MC is not soluble in water at temperatures above its LCST. The remainder of the water, which was at ambient temperature, was added to the aforementioned MC solution as it was stirring. The solution temperature was then rapidly lowered to 0 °C and agitation continued for an additional 15 min. HA powder was then added to the MC solution, and the mixture was stirred for another 10 min. Finally, the NP suspension was added to the hydrogel solution to yield a final concentration of 10 wt.%. This mixture was then incubated overnight at 4 °C prior to experimentation.

### Rheological characterization

An Anton Paar MCR-501 rheometer equipped with a cone-and-plate geometry having a cone angle and diameter of 4° and 25 mm, respectively, was used to assess the rheology of the formulations. The edges of loaded samples were also coated with low-viscosity silicon oil in order to minimize dehydration. Strain and amplitude sweep tests were performed prior to rheological testing in order to identify the ranges of strain and frequency associated with the linear viscoelastic regime. The temperature for sol-gel transition of the formulations was identified by performing small amplitude oscillatory shear (SAOS) experiments by monitoring the crossover of storage (*G*′) and loss (*G*″) moduli at various temperatures [21]. The temperature was increased by a rate of 1 °C/min, while the frequency was set at 1 Hz. The shear-thinning behavior of the formulations was investigated by measuring their viscosities as a function of shear rate, which ranged between 0.01 and 100 s^−1^. Temperature sweeps were performed on three distinct samples of the same composition in order to confirm reproducibility of the results.

### Factorial design of experiments for optimization of hydrogel composition

A sol-gel transition temperature of 32 °C was desired for the formulation. Previous studies on the gelation characteristics of NP-laden hydrogels revealed that the composition of the hydrogel—in particular, the concentration of the precursors and diameter of the NPs—yielded the greatest influence on gelation [22, 23]. As a consequence, the effect of varying the concentrations of HA and MC (*X*_HA_ and *X*_MC_, respectively) and the average diameter of the NPs (*X*_NP_) on the sol-gel transition temperature were systematically investigated in a three-factor, three-level factorial design. The NPs used in the factorial design are composed of carboxy-functionalized poly(styrene). The three levels of each factor were coded as − 1, 0, and 1. The values for coded and actual variables are presented in Table 1. The levels coded as − 1 and 1 correspond to the lowest and highest feasible values at which a particular factor can be set within the system. Since the intended time window for application of this formulation is the patient’s bedtime, the lowest and highest values for *X*_HA_ and *X*_MC_ were determined by assessing of the hydrogel’s degradation rate in STF, and all composites with these constitutions degraded in STF in ~ 8 h. On the other hand, 0 corresponds to either the average of the two extreme values or an intermediate value that is constrained by material availability. The latter is especially relevant for *X*_NP_. The − 1 and 1 levels for NP diameters were selected based on prior reports about the optimal size for corneal penetration and lack of irritancy [24]. Since carboxy-functionalized poly(styrene) bearing an average diameter of 125 nm are not readily available, the 0 level was set at 100 nm, which is sold commercially.

**Table 1:** Coded levels and actual values of the factors investigated in the factorial experiment

Coded variable	Actual variable	Coded levels of factors		
		− 1	0	+ 1
		Actual values of factors		
*X*_HA_	HA concentration (wt.%)	0.5	1	1.5
*X*_MC_	MC concentration (wt.%)	0.5	1	1.5
*X*_NP_	NP size (nm)	50	100	200

The sol-gel transition temperature, which is the dependent variable, was measured using a cone and plate rheometer as described previously. The function *fac.design(*) embedded in the *DoE.base* package of R was used to generate a random order of runs for the 27 experiments. Each experiment was conducted three times, and the effects and interactions between the independent variables were also obtained using R. The significance of the variable effects as well as their interaction effects was evaluated by analysis of variance (ANOVA) for each parameter. Results with a *P* value less than 0.5 were deemed to be statistically significant. Unlike conventional factorial designs, which facilitate identification of an optimal output, we sought to identify an output—not necessarily an optimum point—with a specific characteristic, namely a sol-gel transition temperature of ~ 32 °C. Since this output is not an optimum point, there are many possible combinations that can yield the desired characteristic. The impact of each variable on the output was also predicted using R in order to identify a formulation that gels at 32 °C. Furthermore, some of the 27 experiments were repeated (three replicates) using poly(ethylene glycol) (PEG) NPs. We observed that the composition of the NP did not exert a statistically significant influence on the sol-gel transition temperature. The results of the factorial design informed the optimization of the methodology for synthesis of the drug-loaded PEO-b-PLA NPs.

### Biosynthesis of CBGA

We genetically engineered the bacterium *E. coli* to synthesize CBGA. Genes encoding the rate-limiting enzymes of the native non-mevalonate pathway—*dxs*, *ispD*, *ispF*, and *idi*—were overexpressed on a medium-copy pTrc plasmid under the control of an isopropyl β-d-1-thiogalactopyranoside (IPTG)-inducible *trc* promoter. A strong ribosomal-binding site was employed to drive translation of the transcripts and the plasmid also bears a chloramphenicol selection marker. This plasmid is hereinafter referred to as pTrc-MEP. Overexpression of the aforementioned enzymes enhances production of isopentenyl pyrophosphate (IPP) and dimethylallyl pyrophosphate (DMAPP), which condense together to form geranyl pyrophosphate (GPP). The enzyme GPP synthase (GPPS) catalyzes the condensation of IPP with DMAPP.

In the marijuana plant *Cannabis sativa*, GPP then combines with OA to form CBGA. This reaction is catalyzed by an aromatic prenyltransferase called cannabigerolic acid synthase (CBGAS) [25]. The gene expressing CBGAS [26] was purchased from DNA 2.0, and its codons were optimized for expression in *E. coli*. The synthetic construct was supplied on a high-copy plasmid whereon an IPTG-inducible T5 promoter-controlled transcription. We refer to this plasmid as pT5-CBGAS. We also separately cloned the gene for CBGAS as well as an operon comprising the genes that express GPPS and CBGAS onto a low-copy pBAD33 plasmid under the control of an arabinose-inducible promoter. These plasmids are referred to as pAra-CBGAS and pAra-GPPS-CBGAS, respectively, and both bear the chloramphenicol selection marker. Transformants bearing pT5-CBGAS were selected using kanamycin. All the plasmids used in this study are compatible with one another in co-transformed cells and neither IPTG nor arabinose were observed to affect the performance of the cognate promoters of one another in the ranges of concentrations that were evaluated in this study.

We initially transformed *E. coli* BL21 with pT5-CBGAS and confirmed the synthesis of the desired geometric isomer in vitro using total extracted protein. Briefly, the transformed cells were cultured in 3 mL of LB medium at 37 °C until they reached an optical density (OD) of 0.6. Protein production was subsequently induced using 1 mM of IPTG. The induced cultures were maintained at 30 °C for 4 h. The cultures were then harvested in order to prepare cell extracts for in vitro evaluation of enzyme activity. Replicates of the protein extracts were prepared from three distinct culture tubes. Each 300 μL in vitro reaction comprised 0.2 mM OA, 0.4 or 1 mM GPP, 5 mM MgCl_2_ and 64 μg of unpurified protein in a Tris-HCl buffer at pH 8.5. The reactions were allowed to proceed for 1 h at 30 °C. Following completion of the reactions, the metabolites were extracted in ethyl acetate and analyzed using reversed-phase HPLC on Perkin Elmer Flexar instrument. The HPLC was equipped with a Waters Atlantis C_18_ column (5 μm, 4.6 mm × 250 mm), and CBGA was detected using UV light at 270 nm. An isocratic mobile phase of water:acetonitrile 15:85 and 0.01% trifluoroacetic acid (TFA) at a constant flow rate of 1 mL/min and temperature of 40 °C was used. The peak was measured at a wavelength of 270 nm. For an injection volume of 10 μL, the retention time was ~ 9 min.

The concentration of GPP was deliberately varied to confirm the hypothesis that a stoichiometric excess of GPP is required for the synthesis of CBGA. Synthesis of the E-isomer of CBGA was confirmed by ^1^H-NMR. *E. coli* was then co-transformed with pTrc-MEP, and either pT5-CBGAS or pAra-CBGAS or pAra-GPPS-CBGAS and the production of CBGA were evaluated in vivo. This experiment was replicated three times. In each instance, 5 mL cultures were propagated until they reached an OD of 0.6. The cultures were then induced with 10 mM arabinose and 0.05 mM IPTG and fed with 0.5 and 0.1 mM of GPP and OA, respectively. The cultures were grown overnight for 30 °C, after which the fermentation broth was separated from the cell mass by filtration. CBGA production was then quantified through HPLC. Fifty-milliliter-scale production of CBGA for use in the formulations was subsequently undertaken for 3 days using the most productive of the co-transformed *E. coli* strains, and CBGA was purified from the fermentation broth using flash chromatography. Briefly, the fermentation broth was sonicated to lyse the cells and the liquid medium was filtered to eliminate cell debris. The filtrate was vigorously contacted with ethyl acetate and the organic extract was then dissolved in DMSO for purification using a Reveleris X2 flash chromatography unit. A water-methanol gradient was used to separate CBGA using a SRC C18 column.

### Preparation and characterization of the drug-loaded PEO-b-PLA nanoparticles

The CBGA-loaded PEO-b-PLA NPs were synthesized by nanoprecipitation [27, 28]. Briefly, 10 mg of CBGA and 50 mg of PEO-b-PLA were dissolved in either 5 mL or 10 mL of ethyl acetate. The mixtures were then either sonicated and added dropwise to 100 mL of rapidly stirred water at room temperature or added to water as is, and the solutions were left uncovered for 3 h to facilitate evaporation of ethyl acetate and NP formation. The NPs were then extracted by ultrafiltration using Amicon Ultra-15 centrifuge filters. The filters have a MW cut-off of 30 kDa and were centrifuged at 4000×*g* for 8 min. The concentrated NP suspension was then washed with water and re-centrifuged.

The particle size and polydispersity index of the drug-loaded NPs were measured with a Malvern Zetasizer Nano ZS at 25 °C and at a scattering angle of 90°. Morphological evaluation was performed using a Hitachi S-3000N Scanning Electron Microscope. Briefly, a small volume of the nanoparticle suspension was placed onto a bare aluminum stub and allowed to air dry. The sample was then sputter-coated with 5 nm of gold-palladium alloy. A working distance of 5.0 mm and an accelerating voltage of 5.00 kV were used to image the sample. The NP size distribution and sphericity was also confirmed by estimating the size of 20 randomly selected particles in micrographs of six different regions within the samples using ImageJ. The encapsulation efficiency of the NPs was determined through HPLC quantification of sonicated samples of 1 g of dried NPs in 1 mL of cold acetonitrile in triplicate, as described previously [29].

### Evaluation of the texture of the NP-hydrogel composite

The texture of the NP-laden hydrogel was surveyed using atomic force microscopy (AFM). Measurements were made using a Vecco Multimode 8 Scanning Probe Microscope. Ten microliters of the formulation was spotted on freshly cleaved mica and spread over the surface to form a thin film. The film was allowed to dry under air and then immediately analyzed. Images of a 5-μm-×-5-μm section were captured in AC tapping mode with a tip velocity of 9.8 μm/s, a loop gain of 8, and a scanning speed of 0.977 Hz. The experiment was repeated three times using distinct samples.

### Assessment of in solutio release

Forty microliters of the formulation was injected into a dialysis cassette. The volumetric capacity of the unit is 100 μL, and the cassette was subsequently incubated in 4 L of STF at 32 °C under constant stirring. Ten microliters of the cassette’s contents were withdrawn at intervals of 6 h, and the samples were analyzed using HPLC. The dialysis was repeated three times.

### Corneal penetration study

The delivery of CBGA through the cornea was assessed using freshly excised porcine eyeballs provided by the Centre of Comparative Medicine at the University of British Columbia. The use of a complete Franz diffusion cell, which is how penetration of drugs through tissue is typically evaluated, was avoided owing to the impact on corneal swelling on reproducibility of the data [30]. The eyeballs were obtained with the eyelids intact, which maintains the integrity of the cornea, prevents the hydrogel and ocular surface from drying, and also ensures that the experiment faithfully approximates in vivo conditions. Each eyeball was placed on a concave construct made of plasticine clay, and the constructs were lined with cling film in order to prevent the eyeballs from rolling. The donor compartment of a Franz diffusion cell was subsequently placed atop the cornea and secured using cling film. Forty microliters of the formulation was injected into the donor compartment, and the same concentration of CBGA in mineral oil was used as a control. The entire setup comprising the eyeball atop the plasticine clay holder was immersed in a water bath at 32 °C. With the exception of the donor compartment and a small area surrounding it, the eyeball was entirely submerged in water. Remnants of the formulation were scrapped off the ocular surface after 4 h, and the surface was washed with STF. The cornea and leans were subsequently dissected and separately digested in 1 mL of methylene chloride at 60 °C for 4 h. The tissue samples were then centrifuged, and the CGBA content in the supernatant was analyzed using HPLC. The entire experiment was repeated three times for the experimental and control formulations.

## Results and discussion

### Rheological optimization of the NP-laden hydrogel

**Fig. 1:**
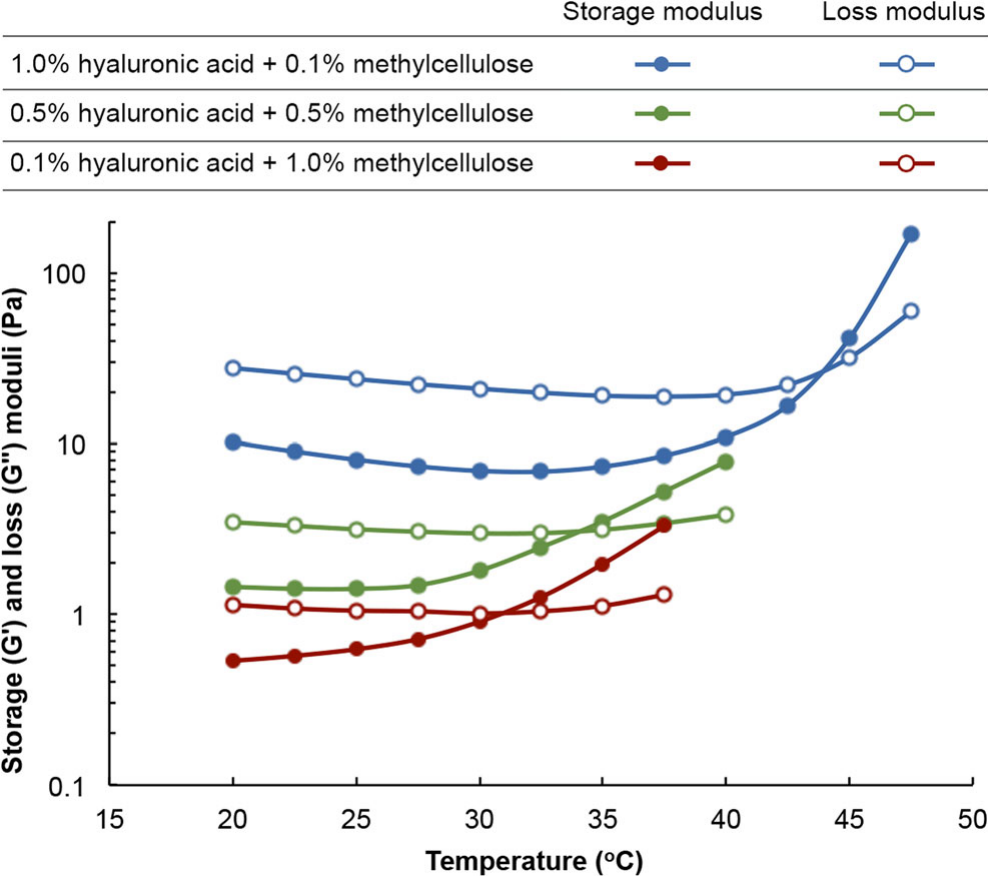
Storage and loss moduli as a function of temperature for hydrogels with different weight percent of HA and MC. All experiments were performed at 1 Hz using a rotational rheometer (MCR-501 of Anton Paar) equipped with a cone-and-plate geometry. Each composition was tested three times, and the standard deviation was estimated to be < 0.03 Pa or < 1.5%. There is no variation in the ordinate variable since the temperature sweeps are electronically controlled

Topical eye drops are the most common dosage form for delivering ophthalmic pharmaceuticals to their site of action [31]. Being liquid, they are easy to manufacture and ship, are relatively easy to apply, and do not negatively affect vision in the manner ointments do. However, as is commonly experienced, very little of the liquid that is delivered to the ocular surface remains there long enough to deliver a sizable dose. Even in instances when the contact time is increased, only a fraction of the drug load in the drop actually penetrates the cornea. It is estimated that < 5% of the drug load in eye drops penetrates the cornea [5, 6]. For anti-neuropathic drugs that are intended to target retinal ganglion cells at the base of the retina, it is safe to assume that only an infinitesimal amount, if at all, of the drug load reaches its site of action. To this end, a formulation that retains the beneficial traits of topical eye drops but eliminates their drawbacks could achieve targeted and deep delivery of ophthalmic drugs. In order to increase contact times yet allow its application as an eye drop, we sought to optimize the composition of the formulation in order to switch its rheological behaviour between thixotropy, wherein its viscosity decreases under shear strain, and temperature-dependent rheopexy, wherein its viscosity increases with temperature. Previous studies have demonstrated that manipulating the concentrations of the formulation’s constituent polymers, HA and MC, and the size of the NPs can achieve switchable behaviour between thixotropy and rheopexy [22, 23]. Since we desire a sol-gel transition temperature of 32 °C and a degradation time in STF of 8 h, we first established a working range for the concentrations of HA and MC by monitoring the crossover of storage (*G*′) and loss (*G*″) moduli during temperature sweeps for several HA-MC mixtures (Fig. 1). The storage modulus is a measure of the elastic behavior of a material, whereas the loss modulus is a measure of its viscous behavior. When a material is liquid, viscous forces dominate its rheology (*G*″ >> *G*′). However, as the material begins to transition to the solid state, the elastic contributions begin to exceed those of viscosity (*G*′ >> *G*″). We observed that although all the mixtures exhibited thixotropic behavior, the MC majority mixtures transitioned from liquid to solid closer to 32 °C, which can be defined as the gel point or sol-gel transition point (*G*′ = *G*″). However, we also noticed that if the concentration of HA fell to 0.1 wt.%, the mixture degraded slowly in STF. Consequently, we established a lower and upper concentration limit of 0.5 and 1.5 wt.%, respectively, for both polymers.

**Fig. 2:**
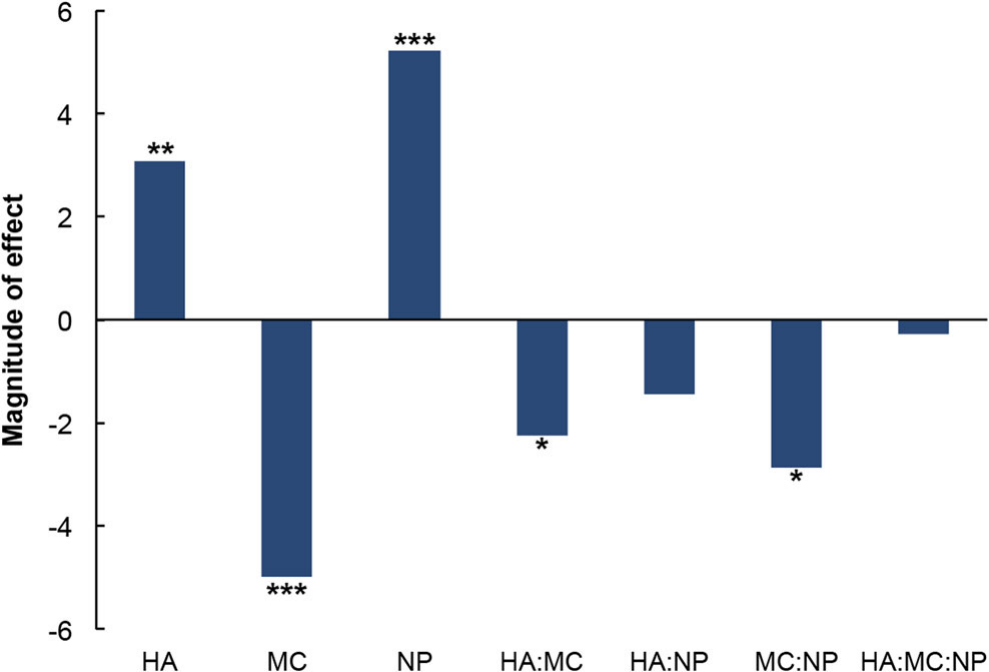
Pareto plot for the evaluation of the effect of HA wt.%, MC wt.%, and NP size and their interactions on the sol-gel transition temperature of NP-laden HA-MC composites (statistical significance codes: ***P = 0; **P = 0.001; *P = 0.01)

We then systematically assessed the influence of polymer concentrations and NP size on the rheological behavior of NP-laden hydrogels in a 3^3^-factorial design. To permit assessment of the independent variables at three levels, we used carboxy-functionalized poly(styrene) NPs instead of amphiphilic NPs. Although the latter are ideal for corneal penetration and transport of lipophilic molecules within the eye, the former are available in defined sizes. We hypothesized that the sol-gel transition temperature of NP-hydrogel composites is more sensitive to the size of the NPs as opposed to their composition. However, since composition and size of the NPs are correlated, we opted to initially investigate the influence of size of the NPs on the sol-gel transition temperature prior to optimizing the composition of the NPs to meet the desired size requirements. This approach ensures that the solution space for optimization of the rheological characteristics and properties of the NP can be interrogated comprehensively in a feasible manner. The Pareto plot for the complete 3^3^-factorial study (Fig. 2) reveals that the concentration of MC and the size of the NPs impact the rheology of the composite in contrasting ways. Specifically, increasing the MC content of the composite reduces its transition temperature, whereas composites containing larger particles undergo sol-gel transitions at higher temperatures.

This observation is consistent with the expectation that increasing the number of entanglements in the MC chains, which occurs at higher concentrations of the material, decreases the amount of energy required for gel formation and that larger particles hinder MC chain entanglements, which necessitates application of a greater amount of energy for gelation to occur. The Pareto plot also confirms that HA concentration is positively correlated with sol-gel transition temperature of the composite, which is in agreement with previous studies that have reported the “salting out” of MC in HA-MC mixtures and the increase in transition temperatures thereafter [22, 32]. All other effects, which included the interactions between the three independent variables, were determined to not be statistically significant to the sol-gel transition temperature of the composite. Consequently, the polynomial that relates the sol-gel transition temperature of the composite to its influential factors (Eq. 1) was deduced and employed to predict that a composite comprising HA and MC at 1.5 wt and 2.5 wt.%, respectively, and NPs of a diameter of 200 nm will achieve a transition temperature that is approximately 32 °C.

1 $$ T=56.32+3.09{X}_{\mathrm{HA}}-4.98{X}_{\mathrm{MC}}+5.23{\mathrm{X}}_{\mathrm{NP}}-2.25{X}_{\mathrm{HA}}{X}_{\mathrm{MC}}-2.86{X}_{\mathrm{MC}}{X}_{\mathrm{NP}} $$

### Microbial synthesis of CBGA

Glaucoma is characterized by high intraocular pressure, which progressively damages the head of the optic nerve. Justifiably, lowering the intraocular pressure has been an extensively exploited therapeutic strategy against glaucoma. Nevertheless, none of these medications remedy the damage that has already been inflicted to retinal ganglion cells that comprise the head of the optic nerve. Reversing this damage through elicitation of neuroprotective mechanisms could prove to be a valuable complementary strategy in addition to lowering intraocular pressure [33]. In particular, activation of the cannabinoid receptors is a promising approach to elicit neuroprotection [7]. The cannabinoid receptors are a unique class of G-protein-coupled transmembrane receptors that are principally located on the surface of neurons and immune cells [34]. These receptors are also found in the gastrointestinal tract, liver, pancreas, adipocytes, eyes, vascular endothelia, spleen, and the lymph nodes. Activation of the cannabinoid receptors triggers downstream signaling and metabolic pathways that subsequently exert a major influence on synaptic transmission, cellular fate, including neuroprotection, and the body’s immune response. Secondary metabolites synthesized by the marijuana plant *C. sativa* rank among the strongest agonists of the cannabinoid receptors. These molecules are aptly referred to as cannabinoids and are essentially prenylated polyketides that are derived from fatty acid and terpenoid precursors [35]. Presently, cannabinoids are produced commercially by directly extracting them from the marijuana plant. However, not only is the source material inconsistent in composition and quality, but its supply too is prone to seasonal and environmental variations. Chemical synthesis of these molecules is also quite challenging, partly due to isomerism. These considerations have greatly limited the use of cannabinoids as active pharmaceutical ingredients as well as additional investigations into the pharmacology of these compounds.

**Fig. 3:**
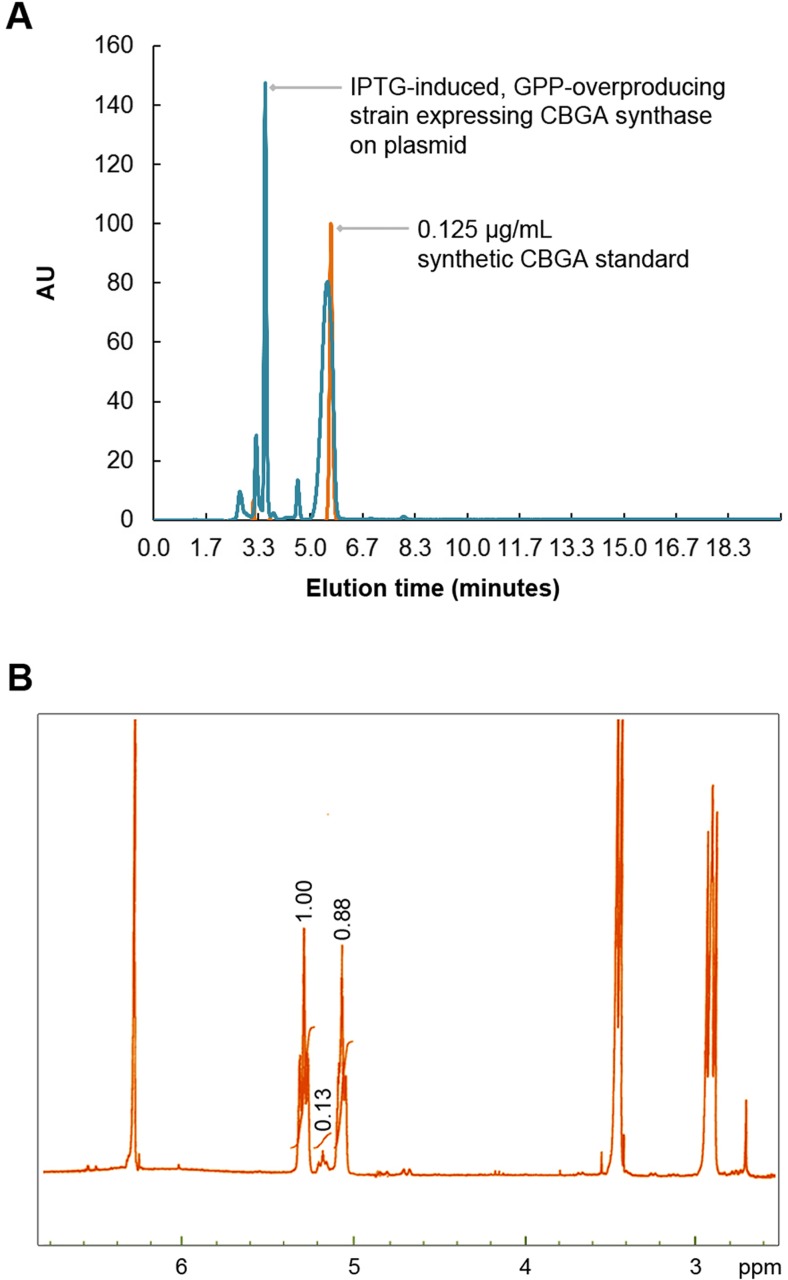
HPLC and ^1^H–NMR analysis of CBGA synthesized in vitro. **a** CBGA was detected at 270 nm. An isocratic mobile phase comprising 15–85% water-acetonitrile solution containing 0.01% TFA was used to elute the metabolites from a C_18_ stationary phase. **b** Preparative HPLC and ^1^H-NMR were employed to confirm the synthesis of CBGA in the in vitro reactions

We have circumvented these obstacles by genetically engineering the bacterium *E. coli* to synthesize a catalog of bioactive cannabinoids. We also investigated cannabinoid biosynthesis in *Saccharomyces cerevisiae*, but we observed that *E. coli* is a much superior and more productive host for production of cannabinoids. One of the molecules that we sought to synthesize was CBGA, which is the product of the condensation between OA and GPP. CBGA is a gateway molecule that is subsequently functionalized to produce other cannabinoids such as tetrahydrocannabinolic acid (THCA) and cannabidiolic acid (CBDA). Although it is not pharmacoactive itself, CBGA is pharmacokinetically similar to other bioactive cannabinoids. In light of the focus of the current study, we have solely reported the results of our study on the biosynthesis of CBGA herein. We confirmed the suitability of *E. coli* as a heterologous host for CBGA biosynthesis by confirming production of the desired E-isomer in vitro (Fig. 3). In order to identify a strain for production of CBGA at higher scales, we subsequently co-transformed *E. coli* with pTrc-MEP and either pT5-CBGAS or pAra-CBGAS or pAra-GPPS-CBGAS and assessed CBGA production in 5 mL overnight cultures. The pTrc-MEP plasmid expresses additional copies of the rate-limiting enzymes of the non-mevalonate pathway that is essential for GPP production.

**Fig. 4:**
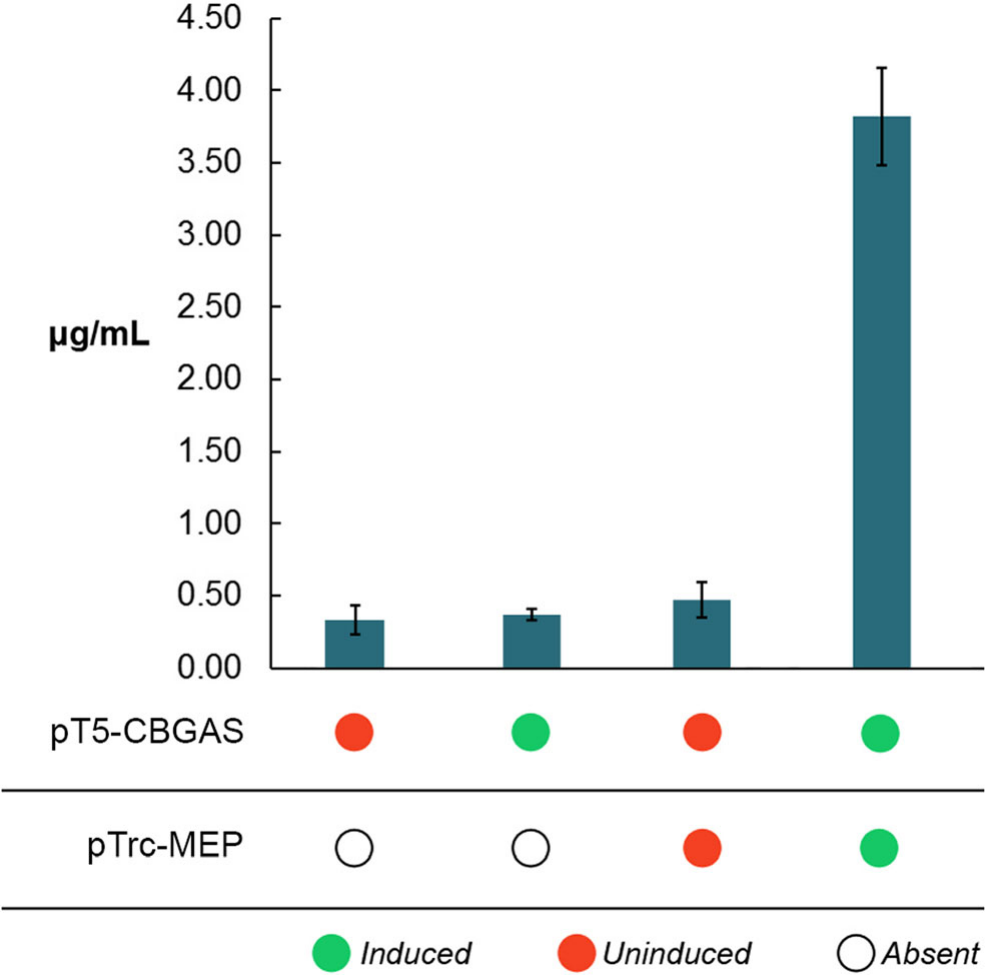
Enhancing GPP flux to the synthesis of CBGA enhances productivity of the strains. CBGA synthase is expressed on a high-copy plasmid under the control of a T5 promoter, whereas the rate-limiting enzymes of the non-mevalonate pathway, whose overexpression is critical for enhancing GPP production, are expressed on a medium-copy plasmid under the control of an IPTG-inducible Trc promoter. The replication and expression of both plasmids is compatible with one another. Error bars represent the standard deviation in the titer of CBGA

Our observations for the pTrc-MEP and pT5-CBGAS co-transformants confirm that supply of GPP is the rate-controlling metabolic flux in the biosynthetic pathway (Fig. 4). In comparison, the pTrc-MEP and pAra-CBGAS co-transformants only achieved a titer of 1.2 μg/mL under the same experimental conditions. Interestingly, overexpression of GPPS did not yield any observable CBGA production.

We speculate that production of GPP over a yet-to-be determined threshold has a detrimental effect on cell health since GPP is toxic to cells at higher concentrations [36]. We subsequently cultivated the *E. coli* strain that co-expresses the pTrc-MEP and pT5-CBGAS in 50 mL cultures for 3 days to produce CBGA for use in the formulations. The average final titer of CBGA in these cultures is approximately 14 μg/mL and the compound is separated via flash chromatography. Each 50 mL batch produces enough material to synthesize in excess of 50 batches of NPs, and the cultures were re-cultivated to meet CBGA requirements as they arose.

### Synthesis and characterization of the CBGA-loaded NPs

**Fig. 5:**
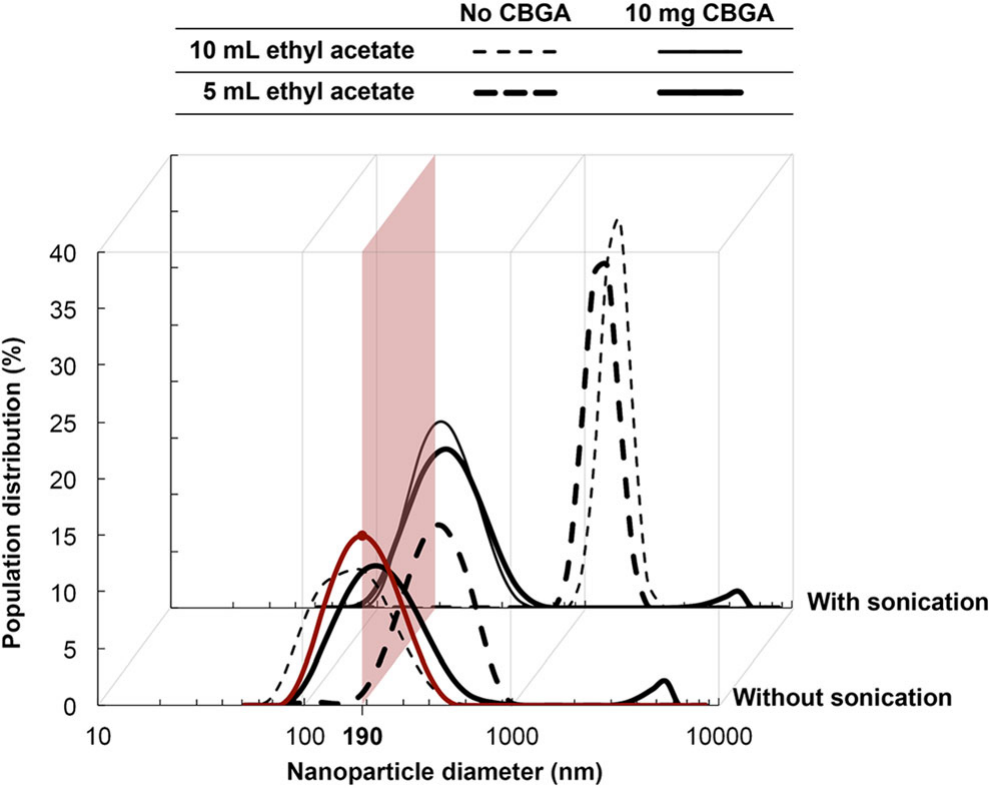
Dropwise addition of a 10-mL solution of CBGA and the co-polymer in ethyl acetate to water produces NPs of the desired size (186 nm) and polydispersity index (0.118). This condition has been highlighted in red in the figure. While sonication is also observed to facilitate dense packing in the core of the NPs, avoiding its use is preferable owing to higher energy demand, particularly at larger scales

We subsequently optimized the nanoprecipitation method in order to synthesize CBGA-loaded NPs having an average diameter of 200 nm and polydispersity index of ~ 0.12. This size meets the rheological requirements of the formulation, does not irritate the cornea, and is ideal for transcorneal penetration [24]. In the absence of pharmacological information regarding optimal dosage for agonists of the cannabinoid receptor, we arbitrarily opted to use 10 mg of CBGA to synthesize a single batch of NPs. Combining an entire batch of NPs with the HA-MC hydrogel should yield a formulation with a theoretical CBGA concentration of 750 μg/mL. We observed that the syntheses involving dissolution of CBGA and PEO-b-PLA co-polymer into either 5 or 10 mL of ethyl acetate, followed by sonication and dropwise addition to 100 mL of water produced particles of the desired size and polydispersity index (Fig. 5).

**Fig. 6:**
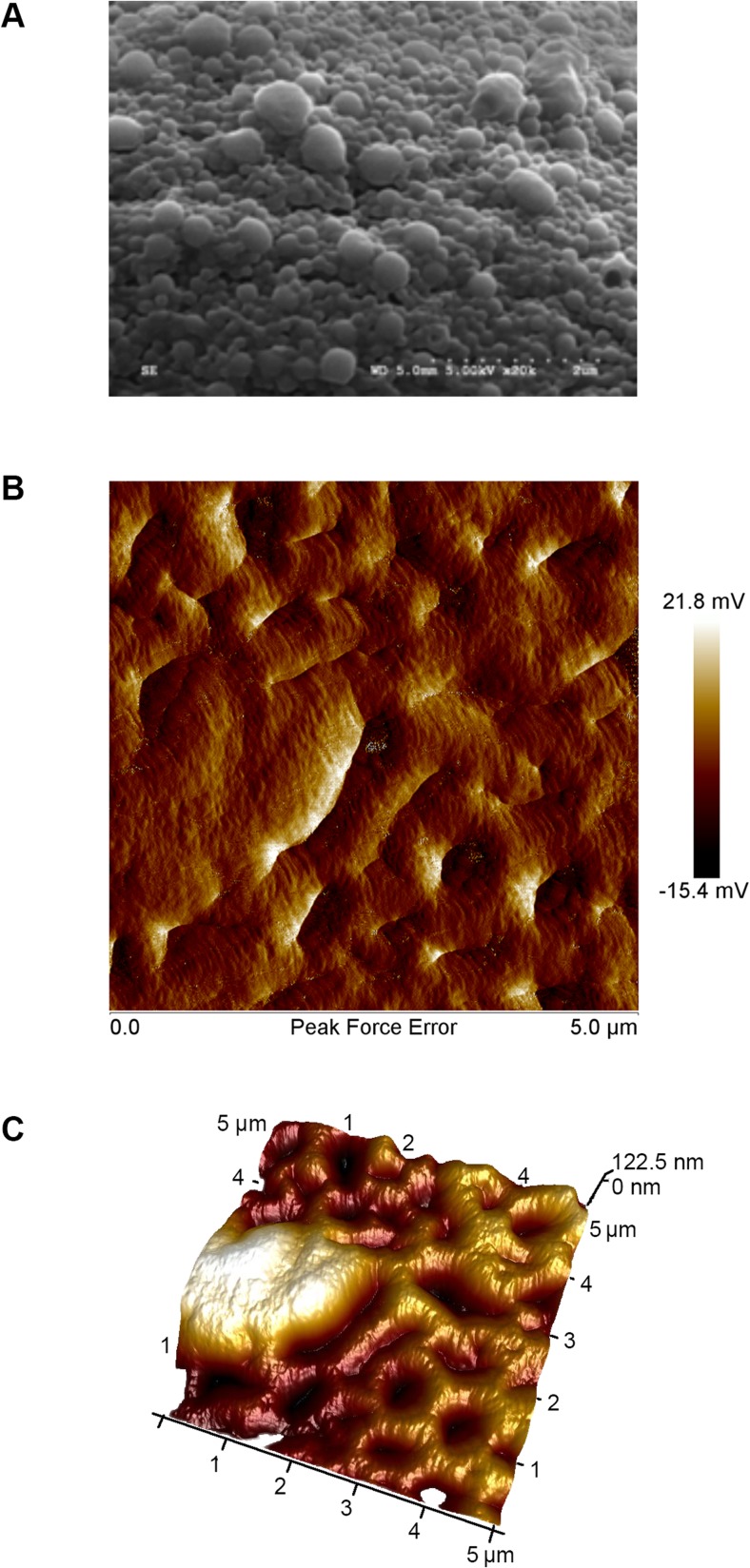
SEM and AFM analysis of the NPs and composite. **a** SEM analysis confirms the formation of spherical NPs with an average diameter of 190 nm. The median and mean particle diameters were determined to be 184 and 196 nm, respectively, and the standard deviation is 6.03 nm. **b**, **c** The formulation exhibits a smooth texture and is uniformly embedded with the NPs

**Fig. 7:**
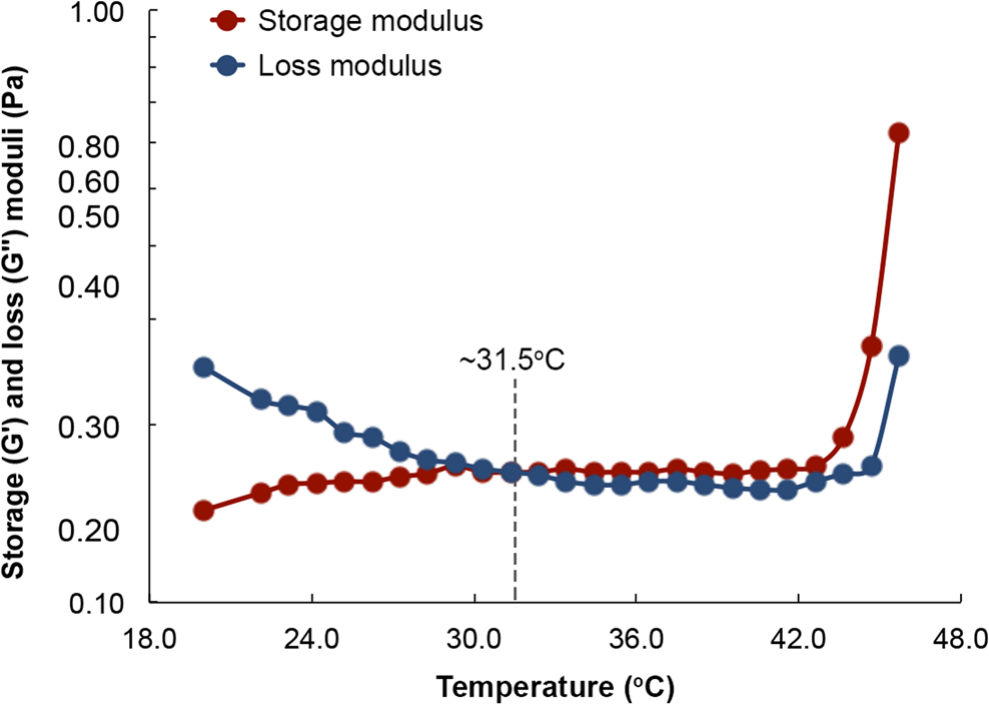
Temperature sweep for the optimized formulation provides a sol-gel transition temperature that is very close to that of ocular surface. The experiment was repeated three times, and the transition temperature was determined to be 31.5 ± 0.2 °C

Likewise, dissolving CBGA and the co-polymer in 10 mL of ethyl acetate in the absence of sonication also yielded particles of the desired size and polydispersity index. Consequently, we employed the method excluding sonication to synthesize the drug-loaded NPs in light of the lower energy demand, should the process mature to the industrial scale. The average diameter and polydispersity index of the NPs produced using the selected method are 186 nm and 0.118, respectively, and the size and spherical morphology of the NPs was also confirmed using SEM (Fig. 6a). Moreover, we observed that loading the NPs with CBGA generally diminished their size, which points to improved packing within the core of the particles, possibly due to co-solubilization. We then concentrated the NP solution using ultrafiltration and combined it with HA and MC to synthesize the NP-hydrogel composite. Images of the surface captured using AFM reveal a smooth texture with uniform embedment of the spherical NPs (Fig. 6b, c). We verified the rheological behavior of the formulation in a final temperature sweep experiment and determined the sol-gel transition temperature to be 31.5 ± 0.2 °C (Fig. 7).

### Performance of the NP-laden hydrogel

**Fig. 8:**
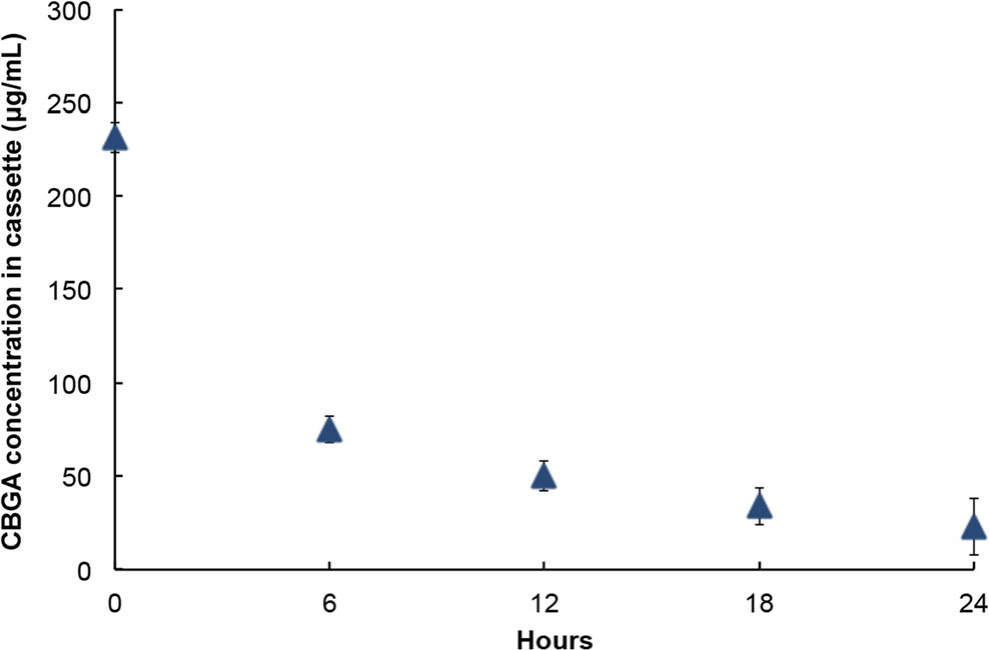
The formulations successfully release CBGA into the bulk aqueous phase and the release kinetics mirrors unsteady state Fickian diffusion, which suggests that the NPs and the formulation behave as intended. Error bars represent the standard deviation in CBGA concentration in the cassette

We estimated the encapsulation efficiency and concentration of CBGA in the formulation via two complementary approaches. In the first method, we dissolved 1 g of dried NPs in 1 mL of cold acetonitrile and quantified the concentration of CBGA in the solution using HPLC. We also estimated the concentration of CBGA in the formulation by analyzing the solution contained in the sample chamber of the dialysis cassette. The concentration of CBGA in the formulation was determined to be 231 ± 26 μg/mL, which translates to an encapsulation efficiency of 30.8 ± 3.5%. We subsequently evaluated the propensity of the formulation to dissolve and release its drug load into aqueous media such as STF and intraocular fluid by quantifying its release kinetics in a dialysis experiment. We observed complete dissolution of the bulk formulation within the first 3 h and sustained release of the drug into the bulk phase over a 24-h period (Fig. 8). It must be noted that we did not assess dissolution of the NPs. However, since the low MW cut-off of the dialysis membrane precludes penetration of the NPs through it, the efflux of CBGA from the dialysis chamber can be solely attributed to sustained dissolution of the NPs and Fickian diffusion of CBGA thereafter. This conclusion is corroborated by the rate of change in the concentration of CBGA in the dialysis chamber. The steep drop between the first two time points can be attributed to dissolution of the formulation and subsequent dilution of CBGA by the STF entering the dialysis chamber. It must be emphasized that this experiment does not perfectly recapitulate the intraocular environment. Nevertheless, the results confirm that the formulation and the constituent NPs behave as intended and our determination of the encapsulation efficiency and release kinetics in the current study provides a frame of reference for the loading and delivery of pharmacologically relevant concentrations of bioactive cannabinoids for the treatment of ganglion cell damage.

**Fig. 9:**
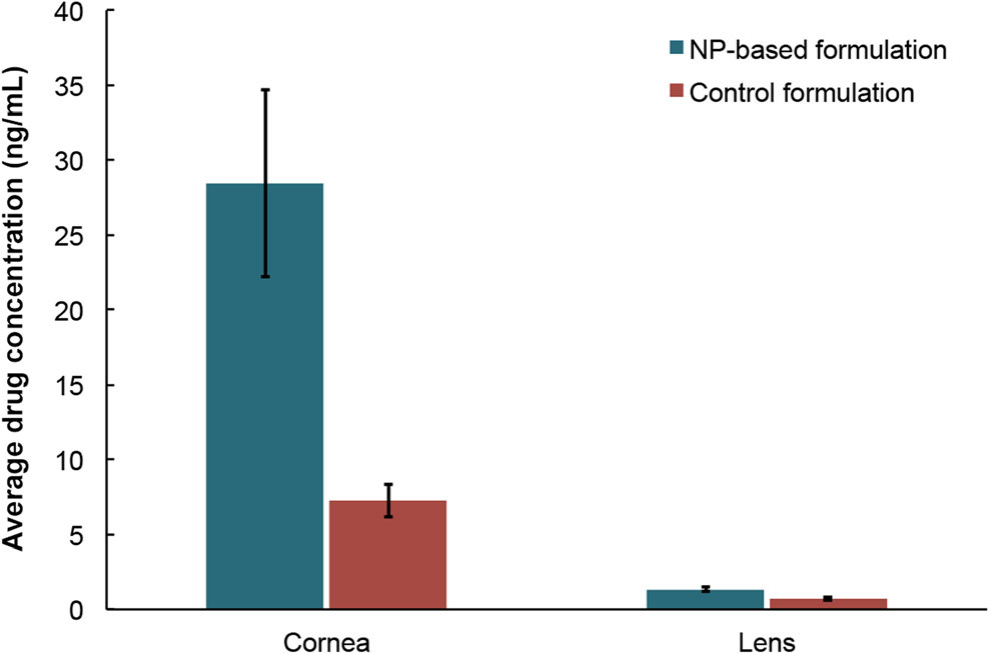
Drug uptake by the cornea and lens after exposing the corneal surface to either the HA-MC hydrogel that is embedded with CBGA-loaded PEO-b-PLA NPs or the control formulation comprising 230 μg/mL CBGA in mineral oil. Error bars represent the standard deviation in CBGA concentration in the tissue

Finally, we also assessed the behavior of the formulation under more physiologically relevant conditions in whole eyeball experiments. Despite being a better experimental system, excised eyeballs do not exhibit lachrymal drainage, which could affect the performance of the formulation. Nevertheless, we observed impressive uptake of CBGA in dissected corneas and lenses after a 4-h treatment (Fig. 9). In fact, our study is the first of its kind to report direct drug uptake by the cornea and lens from a composite NP-hydrogel vehicle. The concentration of CBGA delivered to the cornea and lens by the NP-laden hydrogel is approximately four- and twofold greater, respectively, than the control. Whereas the transport of CBGA through the cornea is purely diffusive in the case of the control, the delivery of CBGA by the amphiphilic NPs is facilitated by diffusion of the NPs themselves. Nevertheless, as was alluded earlier, only ~ 0.015% of the original CBGA load was transported through the cornea, largely due to the absence of lachrymal drainage. In fact, very little of the formulation had dissolved after 4 h. These observations suggest that the formulation could deliver a significantly higher drug load to the intraocular environment under physiological conditions.

## Conclusions

We have successfully developed a stimulus-responsive, in situ-forming, nanoparticle-laden hydrogel for controlled release of poorly bioavailable drugs such as cannabinoids into the aqueous humor of the eye. Using rheology, we systematically optimized the composition of the formulation to achieve a sol-gel transition temperature of 31.5 °C, which is approximately the temperature of the ocular surface. The formulation comprises 1.5 and 2.5 wt.% HA and MC, respectively, and includes CBGA-loaded PEO-b-PLA NPs having an average diameter and polydispersity index of 186 nm and 0.118, respectively. The CBGA used in this study was synthesized using genetically engineered *E. coli*, and we have also established a reliable process to produce CBGA-loaded PEO-b-PLA NPs via nanoprecipitation. HA and MC are biocompatible, highly mucoadhesive, and approved by the FDA as GRAS. However, while HA exhibits concentration-dependent gelation and shear-thinning characteristics [37], gels that solely comprise HA lack structural integrity on account of the high hydrophilicity of biopolymer. On the other hand, while MC is highly viscous and gels in a temperature-dependent manner, it does not gel rapidly enough. When HA and MC are co-formulated together, they interact with one another at the molecular level and yield a material that gels rapidly and can switch its rheology between thixotropy and temperature-dependent rheopexy. Likewise, the amphiphilicity of the PEO-b-PLA NPs is well suited for transporting lipophilic molecules such as cannabinoids through the cornea. Moreover, the size and uniform spherical morphology of the NPs is optimal for transcorneal penetration, efficient dissolution, and reduced irritancy to the ocular tissues. We also observed that incorporating NPs of a specific size further increases the stability of the hydrogel, and this observation is consistent with that of hydrogel formulations that are used for intrathecal injections [22, 38].

When the performance of the formulation was assessed in whole eyeball experiments, we confirmed the improvement in delivery of CBGA by facilitated transport and also observed a 300% increase in transcorneal penetration over the control formulation. In fact, our study is the first of its kind to report direct drug uptake by the cornea and lens from a composite NP-hydrogel vehicle. Nevertheless, there is considerable room for improvement. Only ~ 0.015% of the original CBGA load was transported through the cornea, largely due to the absence of lachrymal drainage. In fact, although the formulation was optimized to dissolve within 8 h under constant lachrymal drainage—and in solutio release studies confirmed that the bulk formulation dissolves completely within the first 3 h and sustains release of CBGA into the bulk phase over a 24-h period—we observed that very little of the formulation had dissolved after 4 h when it was applied onto excised porcine eyeballs. The formulation needs to be assessed in vivo in order to conclusively determine its efficacy in delivering molecules across the cornea and optimize its performance further, including attributes such as optical clarity of the lens. However, the formulation described herein is an excellent starting point and it switches between shear thinning and temperature thickening as intended when it is tested in a rheometer under conditions that are representative of the ocular surface. This result suggests that the formulation could coat the corneal surface by blinking, which implies that the medication could be applied as an eye drop immediately prior to the patient’s bedtime. Liquids are also more desirable than hydrogels since they are easier to manufacture at scale. Our work paves the way for the introduction of novel products targeting ocular diseases to the market.
